# Elevational Constraints on the Composition and Genomic Attributes of Microbial Communities in Antarctic Soils

**DOI:** 10.1128/msystems.01330-21

**Published:** 2022-01-18

**Authors:** Nicholas B. Dragone, Jessica B. Henley, Hannah Holland-Moritz, Melisa Diaz, Ian D. Hogg, W. Berry Lyons, Diana H. Wall, Byron J. Adams, Noah Fierer

**Affiliations:** a Department of Ecology and Evolutionary Biology, University of Colorado—Boulder, Boulder, Colorado, USA; b Cooperative Institute for Research in Environmental Science, Boulder, Colorado, USA; c Department of Natural Resources and the Environment, University of New Hampshire, Durham, New Hampshire, USA; d Department of Geology and Geophysics, Woods Hole Oceanographic Institution, Falmouth, Massachusetts, USA; e Department of Applied Ocean Physics and Engineering, Woods Hole Oceanographic Institution, Falmouth, Massachusetts, USA; f Canadian High Arctic Research Station, Polar Knowledge Canada, Cambridge Bay, Nunavut, Canada; g School of Science, University of Waikato, Hamilton, New Zealand; h School of Earth Sciences, The Ohio State University, Columbus, Ohio, USA; i Byrd Polar and Climate Research Center, The Ohio State University, Columbus, Ohio, USA; j Department of Biology, Colorado State University, Fort Collins, Colorado, USA; k School of Global Environmental Sustainability, Colorado State University, Fort Collins, Colorado, USA; l Department of Biology, Evolutionary Ecology Laboratories, Brigham Young University, Provo, Utah, USA; m Monte L. Bean Museum, Brigham Young University, Provo, Utah, USA; California State University, Northridge

**Keywords:** Antarctica, microbial ecology, soil microbiology, soils

## Abstract

The inland soils found on the Antarctic continent represent one of the more challenging environments for microbial life on Earth. Nevertheless, Antarctic soils harbor unique bacterial and archaeal (prokaryotic) communities able to cope with extremely cold and dry conditions. These communities are not homogeneous, and the taxonomic composition and functional capabilities (genomic attributes) of these communities across environmental gradients remain largely undetermined. We analyzed the prokaryotic communities in soil samples collected from across the Shackleton Glacier region of Antarctica by coupling quantitative PCR, marker gene amplicon sequencing, and shotgun metagenomic sequencing. We found that elevation was the dominant factor explaining differences in the structures of the soil prokaryotic communities, with the drier and saltier soils found at higher elevations harboring less diverse communities and unique assemblages of cooccurring taxa. The higher-elevation soil communities also had lower maximum potential growth rates (as inferred from metagenome-based estimates of codon usage bias) and an overrepresentation of genes associated with trace gas metabolism. Together, these results highlight the utility of assessing community shifts across pronounced environmental gradients to improve our understanding of the microbial diversity found in Antarctic soils and the strategies used by soil microbes to persist at the limits of habitability.

**IMPORTANCE** Antarctic soils represent an ideal system to study how environmental properties shape the taxonomic and functional diversity of microbial communities given the relatively low diversity of Antarctic soil microbial communities and the pronounced environmental gradients that occur across soils located in reasonable proximity to one another. Moreover, the challenging environmental conditions typical of most Antarctic soils present an opportunity to investigate the traits that allow soil microbes to persist in some of the most inhospitable habitats on Earth. We used cultivation-independent methods to study the bacterial and archaeal communities found in soil samples collected from across the Shackleton Glacier region of the Transantarctic Mountains. We show that those environmental characteristics associated with elevation have the greatest impact on the structure of these microbial communities, with the colder, drier, and saltier soils found at higher elevations sustaining less diverse communities that were distinct from those in more hospitable soils with respect to their composition, genomic attributes, and overall life-history strategies. Notably, the harsher conditions found in higher-elevation soils likely select for taxa with lower maximum potential growth rates and an increased reliance on trace gas metabolism to support growth.

## INTRODUCTION

Not all of Antarctica is covered by ice. Ice-free surfaces in Antarctica represent >54,000 km^2^ (∼0.5%) of the total land area of the continent, and most of these ice-free areas are located >5 km from the coast (1). These inland soils can vary in age, from incipient soils that were recently covered in ice to soils that have been ice-free and developing in place for thousands of years or even longer (2). The environmental conditions and geochemical characteristics of Antarctic soils can be highly variable (3–5). However, nearly all of these soils developed under extremely cold and dry conditions—some of the coldest and driest conditions on Earth (6). Most Antarctic soils have extremely low organic carbon concentrations (7, 8), and the nearly complete absence of liquid water can lead many Antarctic soils (particularly those at higher elevations) to accumulate high concentrations of salts over time (2). These salts include nitrate (NO_3_^−^), sulfate (SO_4_^2−^), perchlorate (ClO_4_^−^), and chlorate (ClO_3_^−^) salts derived from atmospheric deposition and chemical weathering (2, 3, 9). Despite extremely challenging conditions, Antarctic soils can harbor diverse and active microbial communities (4, 10).

Which microbes can persist in Antarctic soils and how they are able to tolerate the challenging environmental conditions have long been of interest to scientists (4, 5, 10–12). From this previous work, we know that soil microbial communities found in Antarctica are distinct from those in more temperate ecosystems—distinct with respect to both their taxonomic compositions and their genomic attributes (11–14). Antarctic soils are typically dominated by members of the bacterial phyla *Actinobacteria*, *Firmicutes*, *Bacteroidetes*, and *Proteobacteria* (5, 10). Although these broader taxonomic groups also occur in more temperate soils (15), the specific bacterial taxa and lineages found in Antarctic soils are distinct and often commonly found only in Antarctic soils or other hyperarid environments (16).

Antarctic soil prokaryotes (bacteria and archaea) not only are taxonomically unique but also have specific adaptations for life in Antarctica (12, 14). These adaptations can include those related to osmoregulation and psychrophily that allow microbes to maintain homeostasis and survive Antarctic conditions (6, 13). Microbial communities in Antarctica also use a variety of metabolic pathways to survive in the resource-limited environments typical of most Antarctic soils (17–19). For example, there is accumulating evidence that the metabolism of atmospheric trace gases (including CO, H_2_, and CO_2_) is a key metabolic strategy used by microorganisms in Antarctica and other hyperarid environments to fix carbon and generate energy (20, 21).

Despite the distinct nature of Antarctic soil prokaryotic communities, they are not homogeneous. Previous studies, using both cultivation-dependent and cultivation-independent approaches, have documented a high degree of variance in the composition of Antarctic soil prokaryotic communities (4, 22). While this variability can be attributed to a range of soil and site factors, some of the more important factors shaping the composition of Antarctic soil prokaryotic communities can include temperature, water availability, soil pH, and soil salt concentrations, recognizing that many of these variables often covary across Antarctic landscapes, with drier soils at higher elevations often having higher pHs and higher salt concentrations (23, 24).

The relatively low diversity of most Antarctic soil microbial communities and the pronounced environmental gradients that can be found across sites in Antarctica (even sites located in proximity) make this system well suited for investigating how communities vary across environmental gradients, a core concept in both macroecology and microbial ecology. Likewise, the reduced diversity of Antarctic soil microbial communities, relative to the highly diverse soil communities typical of more temperate environments, makes it possible to relate the taxonomic composition of prokaryotic communities to differences in the functional attributes of these communities (25). Most soil microbes, including those found in Antarctica (16), are difficult to cultivate and study in the laboratory. Fortunately, with advances in cultivation-independent approaches, including shotgun metagenomic analyses, it is now feasible to pair taxonomic- and genomic-based investigations of Antarctic soil microbial communities (26–28). Documenting how the genomic attributes of microbial communities vary across Antarctic soils can contribute important insights into the functional capabilities and adaptations of these unique microbial communities. Based on previous work, we predict that the microbial communities from soil samples collected further inland, i.e., those exposed to more challenging conditions, would be less diverse than those closer to the coast, with regard to both their taxonomic and their functional diversity (23, 24, 29). We also expected that soil communities found further inland at higher elevations would contain more specialized taxa with unique metabolic capabilities, including an increased reliance on trace gas metabolism, that allow them to persist under more resource-limited and challenging environmental conditions.

Here, we analyzed 204 soil samples collected from the Shackleton Glacier region of Antarctica. This region (∼84.5°S to 86.4°S, ∼174.1°W to 177.4°W) includes many ice-free features adjacent to an ∼130-km-long and ∼10-km-wide south-north outlet glacier of the East Antarctic Ice Sheet (EAIS). These soils are highly variable with respect to their ages (amount of time ice-free), geochemistries, and other site conditions (including elevation, temperature, and moisture availability [2]). We analyzed the prokaryotic communities in these soils by coupling a variety of cultivation-independent analyses, including quantitative PCR (qPCR), marker gene amplicon sequencing, and shotgun metagenomic sequencing. Specifically, we used this collection of soil samples and the associated microbial analyses to address two questions: (i) What is the observed variation in the taxonomic compositions and genomic attributes of soil prokaryotic communities across the Shackleton Glacier Region?, and (ii) What soil and site factors explain the observed changes in microbial communities across the Shackleton Glacier region?

## RESULTS AND DISCUSSION

### General characteristics of the soil microbial communities across the Shackleton Glacier region.

The soil samples used for this study represent a wide range of conditions found across the Shackleton Glacier region. For example, the sampling locations ranged in elevation from ∼100 m to over 2,000 m above sea level (ASL), and these soils contained a wide range of concentrations of soluble salts (average, 5.7 × 10^3^ mg/kg; range, 12.6 to 6.7 × 10^4^ mg/kg). Due to the ambient temperatures being well below freezing for most of the year, water availability in these soils is low, and most soils have had prolonged periods of time since the last wetting (mean age since the last wetting, ∼20,000 years) (2). In general, higher-elevation soils were farther from the Ross Ice Shelf, drier (based on the age of the last wetting as estimated by Diaz et al. [2]), and saltier and contained less organic carbon (2, 29). We note that all 10 of the soil and site variables used for downstream analyses (see Materials and Methods) were positively correlated with elevation (*r* > 0.5; *P* < 0.05), although for NH_3_, SiO_2_, and Cl^−^, this correlation was weaker (*r* < 0.25; *P* < 0.05) (see Fig. S1 in the supplemental material). More specific information on the environmental and geochemical properties of these soils was reported previously by Diaz et al. (2), and the raw data can be accessed at https://doi.org/10.5194/bg-18-1629-2021-supplement.

10.1128/msystems.01330-21.1 FIG S1 Correlations between selected soil and site characteristics across the 153 samples used for this study for which all measured variables were available. Pearson correlation coefficients are inset into each scatterplot. While our analyses included soil NH_3_ and SiO_2_ concentrations, they have not been included in this figure as they were not strongly correlated with any other category (but see Data Set S1A in the supplemental material for full soil and site information). Download FIG S1, JPG file, 1.0 MB. Copyright © 2022 Dragone et al. 2022 Dragone et al. https://creativecommons.org/licenses/by/4.0/ This content is distributed under the terms of the Creative Commons Attribution 4.0 International license.

**FIG 1:**
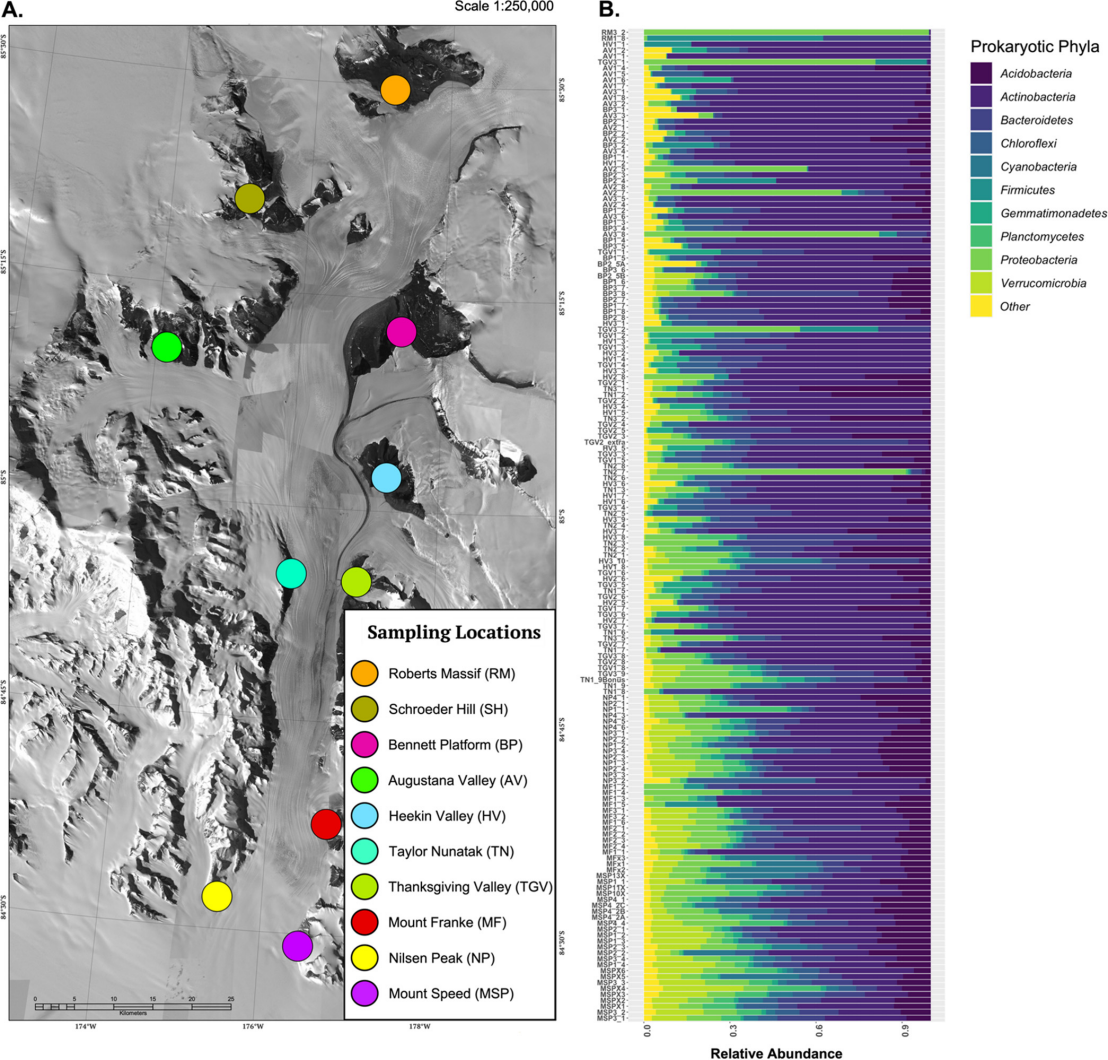
Overview of soil bacterial and fungal community compositions across the Shackleton Glacier region. (A) The Shackleton Glacier region (84°S to 85°S, 174°W to 177°W). Locations of the 10 features where samples were collected are indicated by the colored dots (note that 14 to 26 samples were collected from transects at each of the 10 sampling locations). Map by Mike Cloutier, Polar Geospatial Center (Imagery © 2021 Maxar; reproduced with permission). (B) Relative abundances of the most abundant prokaryotic phyla for each of the 167 samples from which 16S rRNA marker gene sequences were obtained. For panel B, samples are organized from the highest elevation site at the top to the lowest elevation site at the bottom.

**FIG 2:**
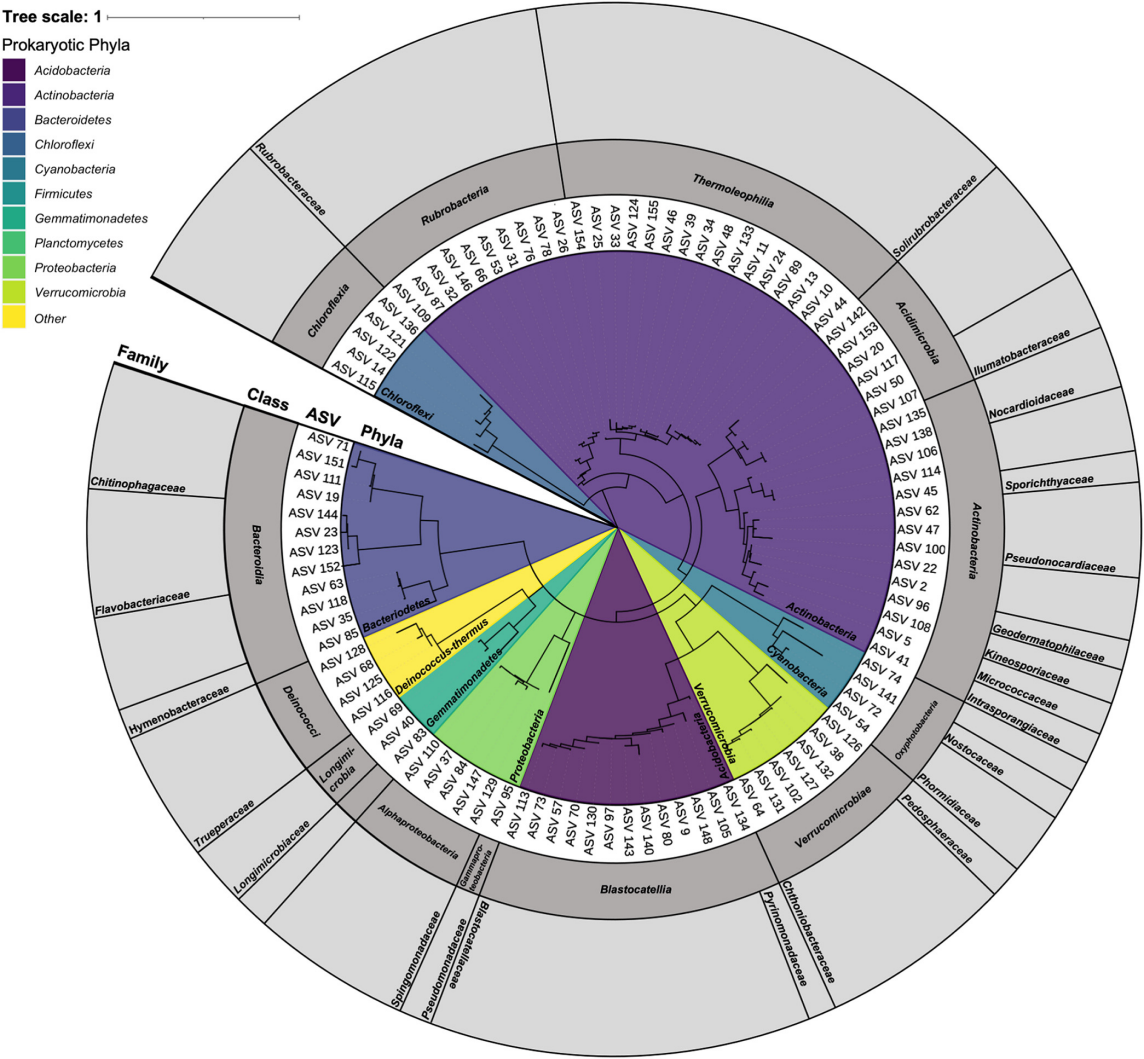
Phylogenetic tree of the taxa from the Shackleton Glacier region of Antarctica based on 16S rRNA gene sequencing (*n* = 167 samples). The ASVs represented in this tree include the top 100 most abundant ASVs that were identified from 16S rRNA gene amplicon sequencing. The inset colors indicate the region of the tree associated with each bacterial phylum, while the gray bars around the ASV labels represent the bacterial order (internal dark-gray ring) and family (external light-gray ring). If no taxonomy is indicated, the ASV is not classified to that taxonomic level (taxonomy, “NA” [not applicable]).

Only 167 of the 204 soil samples yielded a sufficient number of prokaryotic 16S rRNA gene reads from the amplicon sequencing effort for inclusion in downstream analyses (see Materials and Methods) (29). The prokaryotic taxa with the highest relative abundances across these 167 soil samples included those assigned to the bacterial phyla *Actinobacteria*, *Acidobacteria*, *Bacteriodetes*, and *Proteobacteria* (Fig. 1), which make up 46.0%, 11.6%, 10.0%, and 8.1% of the total reads, respectively (Fig. 1). Other phyla identified include *Chloroflexi* (6.2% of the total reads), *Verrucomicrobia* (5.3%), *Gemmatimonadetes* (3.1%), *Cyanobacteria* (3.1%), *Planctomycetes* (2.8%), and *Deinococcus-Thermus* (1.5%). The most abundant amplicon sequence variants (ASVs) across the region were assigned to the families *Solirubrobacteraceae*, *Blastocatellaceae*, *Chitinophagaceae*, and *Rubrobacteraceae* (Fig. 2). Archaeal sequences were found in 60 samples yet made up a maximum of 1.5% of all of the 16S rRNA gene reads per sample (mean, 0.08% of reads per sample), and all were associated with the phylum *Thaumarchaeota*, family *Nitrososphaeraceae*. Prokaryotic richness, the number of distinct 16S rRNA gene phylotypes out of 2,000 reads per sample, averaged 312 ASVs (21 to 853 ASVs), and prokaryotic genome equivalents (a metric of biomass) averaged 2.2 × 10^4^ genome equivalents · g soil^−1^ (Fig. S2).

10.1128/msystems.01330-21.2 FIG S2 Variation and richness of the microbial communities and amounts of microbial DNA in soil samples from the Shackleton Glacier region. (A) NMDS showing dissimilarity in the taxonomic compositions of the microbial communities in the 167 samples based on the 16S rRNA gene sequencing results. (B) Relationship between bacterial richness (number of distinct prokaryotic ASVs out of 2,000 reads per sample) and elevation (meters above sea level). (C) Relationship between the qPCR estimates of prokaryotic DNA concentrations for each soil and elevation. Note that zero values indicate that DNA concentrations were below the limit of detection using our qPCR approach, not that these soils have a complete absence of microbial DNA. In all panels, soil samples are colored by the feature from which they were collected (see the map in Fig. 1A for the locations of sampled features within the Shackleton Glacier region). Download FIG S2, JPG file, 0.5 MB. Copyright © 2022 Dragone et al. 2022 Dragone et al. https://creativecommons.org/licenses/by/4.0/ This content is distributed under the terms of the Creative Commons Attribution 4.0 International license.

The composition of the prokaryotic communities in these soil samples is consistent with the results obtained using similar cultivation-independent analyses of other Antarctic soils, including those from the McMurdo Dry Valley region (23, 24). However, we note that the composition of the microbial soil communities of the Shackleton Glacier region is highly variable, particularly with respect to the relative abundances of major taxonomic groups. For example, *Actinobacteria* and *Chloroflexi* were relatively more abundant in soil samples from the higher elevations (Fig. 1). Most ASVs were detected in only a few soil samples; out of the 8,641 prokaryotic ASVs identified through 16S rRNA gene sequencing, 5,335 were found in <10 soil samples.

### Key drivers of prokaryotic community composition.

The observed differences in the compositions of the prokaryotic communities across the region were best described by a model that included elevation and total salt, perchlorate, and chlorate concentrations (*r* = 0.62; *P < *0.001). Of these variables, elevation alone explained the majority of the dissimilarity (*r* = 0.47; *P < *0.001), with the other variables contributing less to the overall correlation (total salt *r* = 0.11, perchlorate *r* = 0.02, and chlorate *r* = 0.03; *P < *0.05). The importance of elevation to the overall degree of dissimilarity in prokaryotic communities is further supported by Mantel analyses, which showed a reasonably strong correlation between elevation and Bray-Curtis distances across the 167 samples (*r* = 0.45; *P < *0.001). These results suggest that elevation is the most important predictor of the degree of dissimilarity in prokaryotic communities across the Shackleton Glacier region of Antarctica. Elevation also had a reasonably strong influence on soil prokaryotic richness (*r* = 0.56; *P < *0.001) (Fig. S2). As the elevation increased, we observed a steady decrease in prokaryotic richness up to ∼2,000 m. In general, the higher-elevation soils also had the lowest concentrations of prokaryotic DNA, although this correlation was weak (*r* = 0.09; *P < *0.05).

Elevation is unlikely to be the sole factor driving the observed differences in microbial community structure. Instead, these results support previous hypotheses that the soil environments found at higher elevations and further inland exert increasingly strong selective pressures on soil microbial communities (4, 5, 29) by virtue of these higher-elevation soils being saltier, colder, and often drier. The differences in elevation may also be associated with other important variables that we were unable to measure. For example, in Antarctic soils, elevation has been positively correlated with increases in UV radiation, decreases in temperature, and a decrease in available water (increased age of the last wetting estimated from concentrations of water-soluble salts) (2, 5). These variables are difficult to measure in this remote area where visits are short and infrequent, but all of these variables have been shown to have potentially important effects on Antarctic soil communities (2, 6, 29–31).

### Certain taxa are associated with specific soil and site conditions.

**FIG 3:**
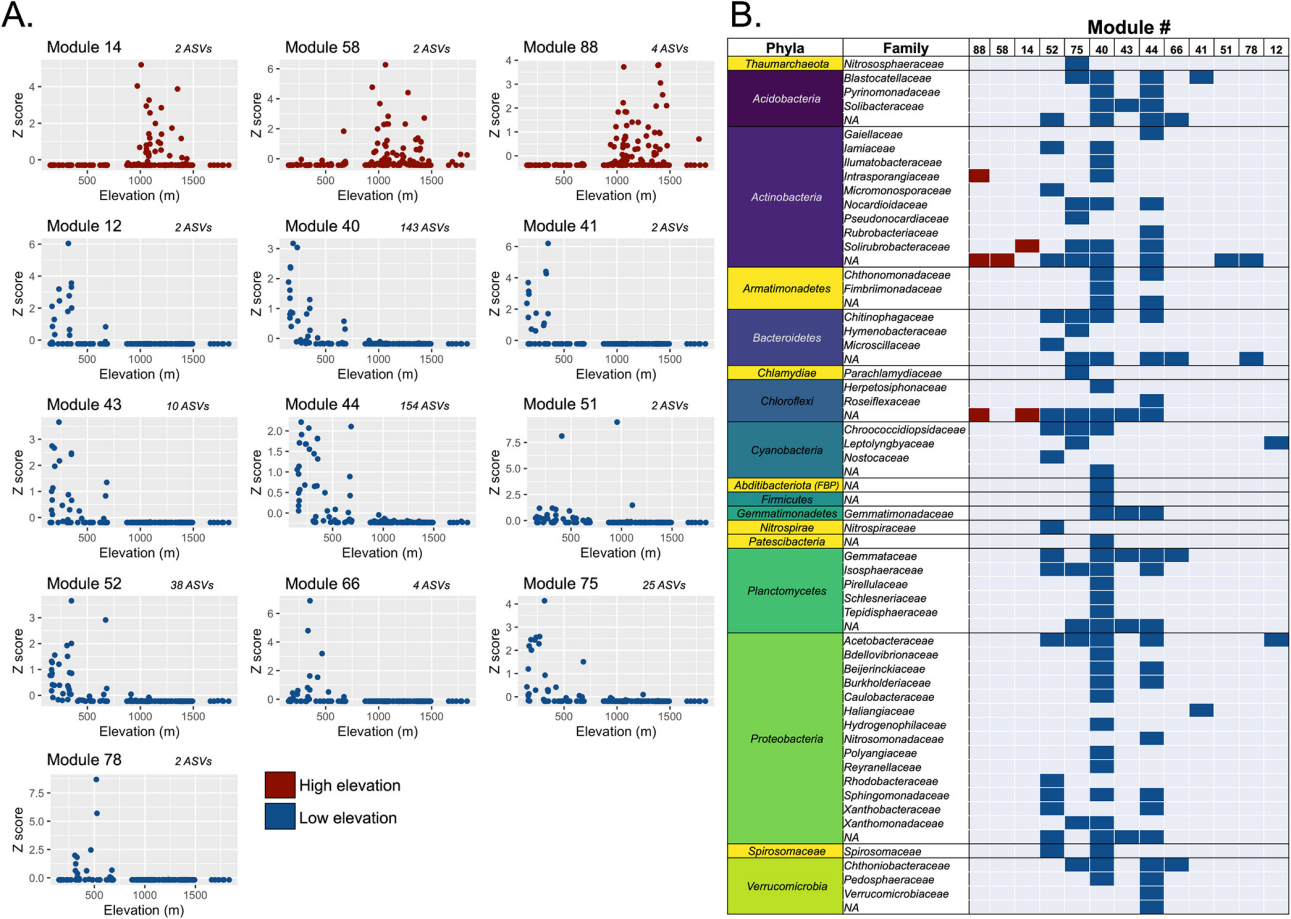
Taxonomic compositions and elevational preferences of 13 prokaryotic modules identified from 167 samples. Modules are groups of ASVs (nearly all bacterial) that were identified as cooccurring based on the results of the network analysis. The modules displayed are modules with distributions best explained by site elevation based on the results of the random forest analysis. (A) Average standardized relative abundances (Z score) plotted against elevation of the 3 high-elevation modules and the 10 low-elevation modules. The numbers of ASVs that are included in each module are listed next to the module number. (B) Phylum- and family-level taxonomic identities of those ASVs associated with each of the 13 modules (3 high elevation and 10 low elevation).

We were able to identify the environmental preferences of 28 of the 88 prokaryotic modules using network analyses and random forest analyses (Table S1). The majority of these (16 of the 28 modules) were most strongly associated with elevation. For this reason, and due to the fact that nine of the remaining modules were predicted by variables strongly correlated with elevation (Table S1 and Fig. S3), we chose to focus the majority of our analyses and interpretation on the taxa assigned to modules associated with elevation. Out of the 16 modules best predicted by elevation, 3 were found to be associated with only the higher-elevation sites (>800 m), 10 were found to be associated with only the lower-elevation sites (<800 m) (Fig. 3), and 3 were found to be associated with mid-elevation sites. There were more ASVs associated with low-elevation modules (average, 38 ASVs; range, 2 to 154 ASVs) than with high-elevation modules (average, 3 ASVs; range, 2 to 4 ASVs), and these “low-elevation” ASVs included representatives from 61 different families and 17 phyla. In comparison, all of the ASVs associated with the “high-elevation” modules consisted of taxa assigned to the phyla *Actinobacteria* (including taxa within the families *Solirubrobacteraceae* and *Intrasporangiaceae*) and *Chloroflexi* (Fig. 3). Together, these results highlight that differences in elevation (or environmental variables associated with elevation) can explain a large portion of the observed variation in the overall community composition (Fig. 1) and the distributions of particular prokaryotic taxa (Fig. 3).

10.1128/msystems.01330-21.3 FIG S3 Environmental preferences of 12 bacterial modules. The 12 bacterial modules that were not best predicted by elevation (see the text for details) are shown. Z scores are the average standardized relative abundances of all of the ASVs associated with each module (range of 2 to 154 ASVs per module). See Data Set S1C in the supplemental material for information on the ASVs associated with each module. Download FIG S3, JPG file, 0.5 MB. Copyright © 2022 Dragone et al. 2022 Dragone et al. https://creativecommons.org/licenses/by/4.0/ This content is distributed under the terms of the Creative Commons Attribution 4.0 International license.

10.1128/msystems.01330-21.8 TABLE S1 Summary of the results of the random forest analyses. A variable was determined to be the most predictive variable if the model explained at least 10% of the variance and if that variable increased the MSE by at least 5% (*P* < 0.05). Information on the ASVs assigned to each module can be found in Data Set S1C in the supplemental material. Download Table S1, JPG file, 0.2 MB. Copyright © 2022 Dragone et al. 2022 Dragone et al. https://creativecommons.org/licenses/by/4.0/ This content is distributed under the terms of the Creative Commons Attribution 4.0 International license.

### Genomic attributes of microbial communities.

**FIG 4:**
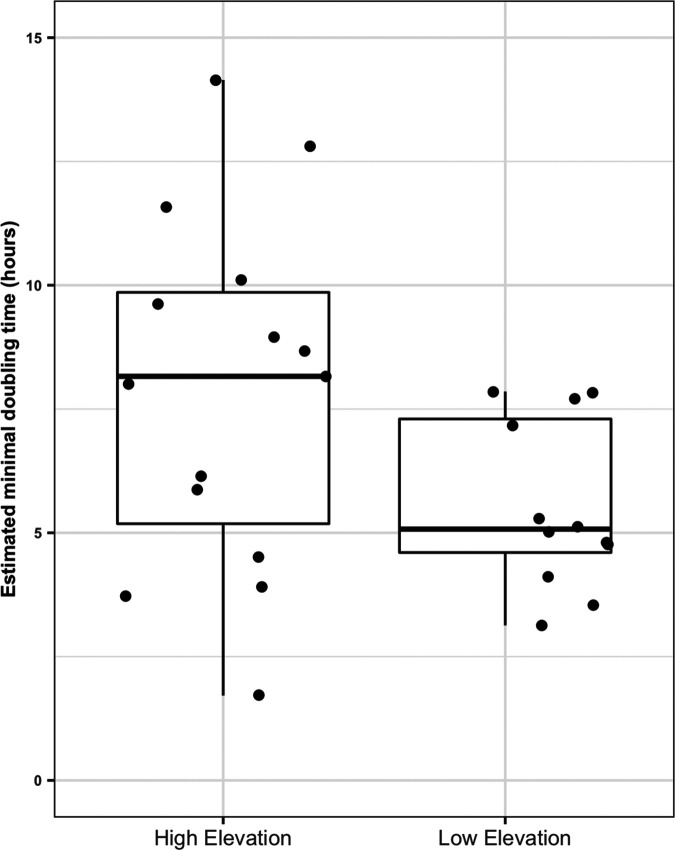
Patterns in estimated doubling times of samples (*n* = 27) across the Shackleton Glacier region. Fifteen samples make up the above 800-m ASL “high-elevation” group, while 12 samples make up the below 800-m ASL “low-elevation” group. Estimated minimum doubling times, as inferred from gRodon (56), were longer (lower maximum potential growth rates) in higher-elevation samples than in lower-elevation samples (*P* = 0.042 by a Mann-Whitney U test). We note that as gRodon was not designed for use on communities from these types of environments, the estimates of minimal doubling times are presented for comparative purposes only, and the exact values should be considered with caution.

By analyzing shotgun metagenomic data obtained from 27 soil samples that were selected from the large sample set (see Materials and Methods), we found that, on average, the communities in the high-elevation samples had longer estimated minimum doubling times (lower maximum potential growth rates) than the communities in the low-elevation samples (*P* = 0.042 by a Mann-Whitney U test) (Fig. 4), although we caution that maximal potential growth rates should be considered estimates for comparative purposes only, not actual growth rates. The dissimilarities in the functional gene profiles were positively correlated with the dissimilarities in the taxonomic composition of the prokaryotic communities (*n* = 27) (*r* = 0.70; *P < *0.001). This suggests that overall taxonomic dissimilarity in communities can be used to predict community-level differences in functional gene composition, a pattern consistent with observations from other metagenomic studies (14, 31). We also found that the number of distinct functional genes (richness) in the high-elevation samples was significantly lower than that in the low-elevation samples (*P < *0.001 by a Mann-Whitney U test) (Fig. S4). Larger numbers of genes were more than twice as abundant at low elevations than at high elevations (6,406 KEGG Objects [KEGGs]) compared to those more abundant at higher elevations than at low elevations (918 KEGGs). Functional gene richness was well correlated with the observed patterns in taxonomic richness across these samples (*n* = 27) (*r* = 0.77; *P < *0.001).

10.1128/msystems.01330-21.4 FIG S4 KEGG gene richness in soils above and below 800 m. Fifteen samples make up the above-800-m “high-elevation” group, while 12 samples make up the below-800-m “low-elevation” group. KEGG gene richness was significantly lower in the higher-elevation samples than in the lower-elevation soil samples (*P < *0.001 by a Mann-Whitney U test). Download FIG S4, JPG file, 0.1 MB. Copyright © 2022 Dragone et al. 2022 Dragone et al. https://creativecommons.org/licenses/by/4.0/ This content is distributed under the terms of the Creative Commons Attribution 4.0 International license.

Functional gene analyses to identify potential functional pathways of interest that may be overrepresented in high-elevation Antarctic soils identified many functional pathways associated with metabolism (49% of the pathways containing high-elevation-associated genes are related to metabolism) (Fig. S5). This identification of high-elevation-associated metabolic genes supports previous work suggesting that microbial communities use a greater variety of genes coding for metabolic pathways in more challenging Antarctic environments (17–19). Of note, the metabolic pathway with the greatest number of genes overrepresented in the higher-elevation soils was the “methane metabolism” pathway (KEGG pathway KO00680). Trace gas metabolisms, including methanotrophy, are important metabolic strategies used by microorganisms in Antarctica and other hyperarid environments to generate energy and fix carbon (19, 20), yet this evidence suggests that the relative importance of these metabolic strategies may increase under more challenging conditions.

10.1128/msystems.01330-21.5 FIG S5 Gene pathways that are relatively more abundant in higher-elevation soils. Shown here are those gene categories with at least 5 genes that were identified as being >2 times more abundant in samples above 800 m across our sample set (comparing 15 soil samples collected from above 800 m and 12 soil samples collected from below 800 m). Download FIG S5, JPG file, 0.8 MB. Copyright © 2022 Dragone et al. 2022 Dragone et al. https://creativecommons.org/licenses/by/4.0/ This content is distributed under the terms of the Creative Commons Attribution 4.0 International license.

**FIG 5:**
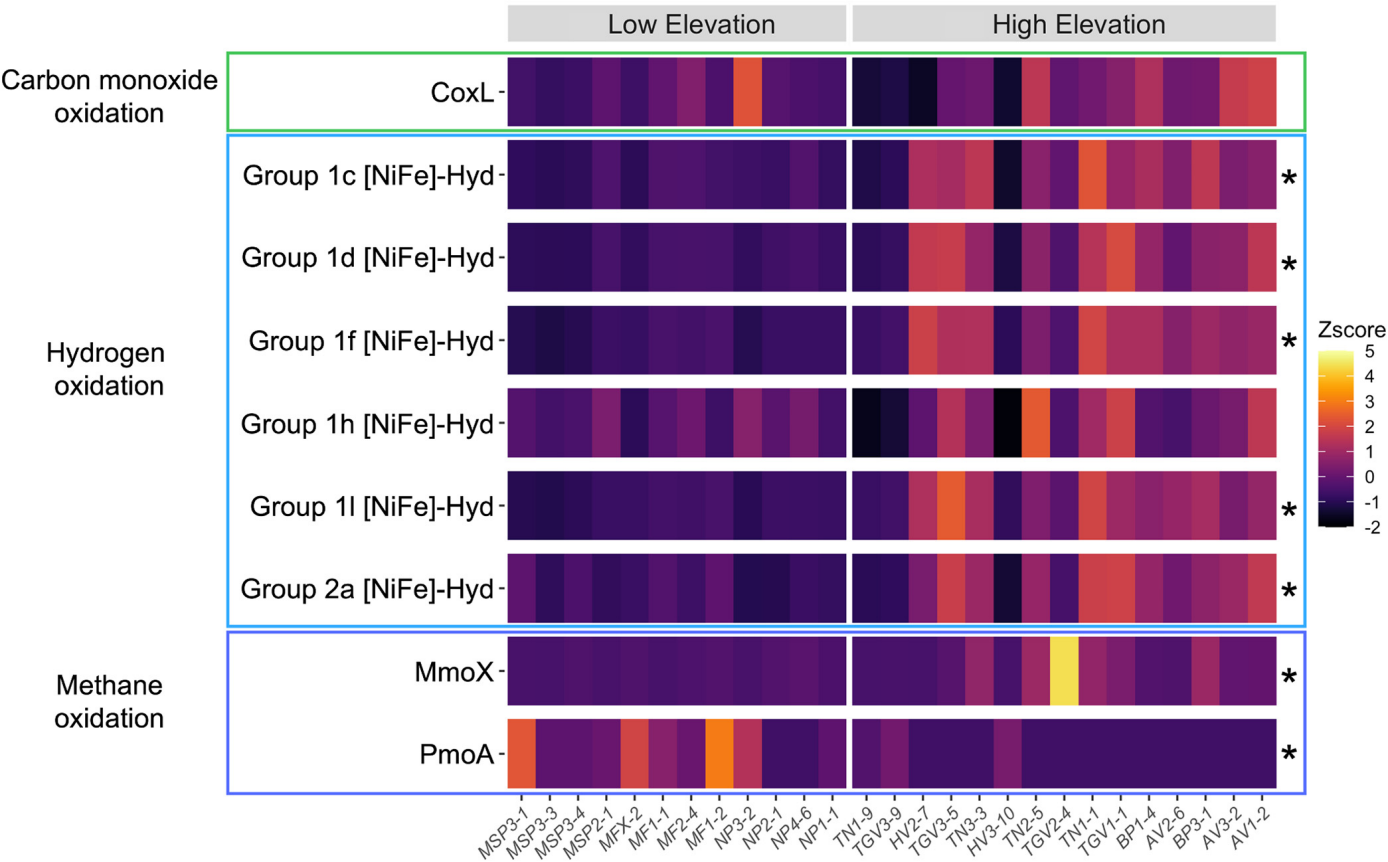
Abundances of nine genes associated with trace gas metabolism across the Shackleton Glacier region. The 27 samples are grouped from lowest elevation to highest elevation and are grouped into high-elevation and low-elevation categories based on whether they were collected above or below 800 m ASL. Groups of genes associated with specific trace gas oxidation pathways are outlined by colored boxes. Z scores were calculated based on the proportional gene abundances, which are presented in Data Set S1D in the supplemental material. Significant differences in gene abundances between the two groups are starred, and the associated statistical information can be found in Fig. S6.

To complement the functional gene analyses described above, which focused only on broader gene categories, we performed more targeted analyses to compare the abundances of genes associated with trace gas metabolism given that this category of functional genes was consistently overrepresented in higher-elevation soils (Fig. S5) and given the potential importance of trace gas metabolism as a strategy for microbial survival in hyperarid systems (32). We were able to identify genes associated with carbon monoxide oxidation, hydrogen oxidation, and methane oxidation in almost all of the samples (Fig. 5). By comparing metagenomes from high-elevation and low-elevation soils, we found that five of the six genes involved in hydrogen oxidation were significantly more abundant in high-elevation soils than in low-elevation soils, as was the gene coding for soluble methane monooxygenase (MmoX) (Fig. 5; Fig. S6). Notably, the most abundant genes related to hydrogen oxidation were those in the recently identified group 1l [NiFe]-hydrogenases (Hyd) (HylL), which have been shown to be the primary catalysts of hydrogen oxidation in cold desert soils in other regions of Antarctica (19). The higher abundance of genes associated with hydrogen oxidation and methane oxidation suggests that trace gas metabolism may be particularly important for sustaining microbial life in higher-elevation Antarctic soils. In contrast, the carbon monoxide dehydrogenase (CoxL) gene was found in almost all of the samples, and particulate methane monooxygenase (PmoA) was significantly more abundant in lower-elevation soils (Fig. 5; Fig. S6).

10.1128/msystems.01330-21.6 FIG S6 Comparison of the abundances of 10 genes associated with trace gas metabolism. High-elevation samples were collected from above 800 m (*n* = 15), while low-elevation samples were collected from below 800 m (*n* = 12). For all comparisons, the significance (*P*) (as determined by a Mann-Whitney U test) is included with the gene name in the title of each plot. The data used to generate these plots can be found in Data Set S4 in the supplemental material. Download FIG S6, JPG file, 0.9 MB. Copyright © 2022 Dragone et al. 2022 Dragone et al. https://creativecommons.org/licenses/by/4.0/ This content is distributed under the terms of the Creative Commons Attribution 4.0 International license.

10.1128/msystems.01330-21.10 DATA SET S1 (A) Locations and environmental/geochemical characteristics associated with the 167 soil samples that passed our sequencing threshold for inclusion in this study (see Materials and Methods for more details). Values that were not recorded for a sample are noted. (B) Table of all ASVs with at least 10 reads in total across the entire data set (8,671 ASVs) recovered through our culture-independent marker gene sequencing of the 16S rRNA gene. The relative abundance of each ASV in each sample is represented as the proportion of total reads assigned to that sample. The taxonomic classification of each ASV and the 16S rRNA sequence for that ASV are also included. (C) Identities and relative abundances of the 885 ASVs assigned to each of the 88 modules identified through network analysis. The relative abundance of each ASV in each sample is represented as the proportion of total reads assigned to that sample. (D) Table of the abundances of the 10 genes associated with trace gas metabolism generated through targeted metagenomic analysis. The relative abundance of each gene in each of the 27 samples is represented as the proportion of total reads assigned to that sample. Download Data Set S1, XLSX file, 7.0 MB. Copyright © 2022 Dragone et al. 2022 Dragone et al. https://creativecommons.org/licenses/by/4.0/ This content is distributed under the terms of the Creative Commons Attribution 4.0 International license.

Our results build on those reported previously in highlighting that trace gas metabolism is likely an important metabolic strategy used by Antarctic soil microbial taxa (19, 20) and support previous work that atmospheric trace gas metabolism, and in particular hydrogen oxidation, is widespread in a variety of soil environments (32, 33). These metabolic strategies may be particularly important in Antarctic soils, where biogeochemical studies suggest that the rate at which soil communities oxidize atmospheric trace gases may be sufficient to sustain their energy needs under certain conditions (20). The differential abundances of these genes across our data set suggest that the importance of certain trace gas metabolisms by communities may vary depending on the environmental or geochemical conditions. We may see more H_2_ oxidation genes in higher-elevation soil communities, for example, because H_2_ occurs consistently in low abundance in the troposphere, with microbes using H_2_ as an energy source to sustain metabolic activity in resource-limited environments (19–21).

### Conclusions.

Microbial communities across the Shackleton Glacier region are highly variable in composition, and this variation is strongly associated with elevation (or environmental variables that strongly covary with elevation). Higher-elevation soils typically had lower biomass, less diverse prokaryotic communities (Fig. S4), and communities with longer minimum doubling times (as estimated from metagenome-based analyses of codon usage bias) (Fig. 4). Likewise, we found that elevation was also associated with differences in the overall compositions of the microbial communities, with the distributions of numerous specific prokaryotic taxa being best predicted from site elevation (Fig. 3). Finally, the genomic attributes of the communities differed across the elevation gradient, with a notable increase in the abundance of genes for trace gas metabolism in higher-elevation soils and the prokaryotic communities found at higher elevations having lower estimated maximum potential growth rates (Fig. 4). The sampled elevation gradient captures a gradient in microbial community composition, with the communities found in the more challenging soil environments at the margins of habitability (29) having distinct life-history strategies and corresponding genomic attributes.

The patterns documented from our cultivation-independent methods would be difficult to infer from studying cultivated isolates, as most culturing studies are able to isolate taxa that represent only a fraction of the microbial diversity found in soil. Despite these limitations, detailed studies of cultivated isolates would make it possible to link the observed distribution patterns of particular taxa with experimental measurements of environmental preferences and tolerances (e.g., measuring growth responses across gradients in temperature and moisture *in vitro*). Likewise, while our analyses shed light on the potential functional attributes of high-elevation and low-elevation communities, the metabolic capabilities of specific taxa remain elusive. An important next step would be to identify which specific taxa harbor particular genes of interest, particularly those genes associated with the metabolism of H_2_, CO, and CH_4_. This could be done by pairing community-level metagenomic analyses (as done here) with detailed analyses of particular metagenome-assembled genomes, as demonstrated previously (19). Integration of community-based and organismal-based ecological, genomic, and trait-based information will provide a more comprehensive understanding of microbial life in Antarctic soils and the adaptations that allow specific taxa to survive in one of the most challenging terrestrial environments on Earth.

## MATERIALS AND METHODS

### Sample collection and characterization.

Soil samples were collected from the Shackleton Glacier region from December 2017 to January 2018. The soil sampling process was described in detail previously by Diaz et al. (2) and Dragone et al. (29). In brief, soil samples were collected from 10 different features running the length of the valley, including a range of elevations (150 to 2,221 m above sea level [ASL]) across a 120-km north-south distance spanning from the Ross Ice Shelf to the Polar Plateau (Fig. 1). Between 14 and 26 soil samples were collected along elevational transects located on each of the 10 features to maximize variation in soil characteristics and soil exposure times (amount of time at the surface and uncovered by glacial ice) at each feature. Soil samples (0- to 5-cm depth) were collected into sterile polyethylene bags using ethanol-cleaned hand trowels. GPS coordinates, photographs of the soil surface, elevation, and other environmental data were collected at the time of soil sample collection. All soil samples were transported to the field camp in insulated coolers where they were frozen at −20°C and remained frozen until they were processed at the University of Colorado in Boulder, CO.

Environmental and geochemical variables associated with each sample were measured as described previously by Diaz et al. (2). For this study, we chose to focus on the following variables: elevation (meters ASL), nitrate (milligrams per kilogram of soil), chloride (milligrams per kilogram of soil), total cations (milligrams per kilogram of soil), total anions (milligrams per kilogram of soil), total salt (milligrams per kilogram of soil), perchlorate (micrograms per kilogram of soil), chlorate (micrograms per kilogram of soil), NH_3_ (milligrams per kilogram of soil), and SiO_2_ (milligrams per kilogram of soil). We focus our analyses on these 10 variables as they are likely the most biologically relevant and were measured for at least 80% of the soil samples (2, 29). See Data Set S1A in the supplemental material for the geochemical data associated with each sample included in this study. All concentration measurements were log transformed prior to downstream analyses. Correlations between these environmental and biogeochemical datum variables were calculated with the R function cor (method, pearson), and correlation matrix plots were visualized using ggpairs (R package GGally) (Fig. S1).

### Cultivation-independent analysis.

DNA was extracted from 204 samples in a laminar flow hood. After mixing 1 g of each soil sample in 1 mL of sterile PCR-grade water, DNA was extracted from a 500-μL aliquot of the soil slurry using the Qiagen (Germantown, MD, USA) DNeasy PowerSoil HTP 96 kit according to the manufacturer’s recommendations. A total of 6 extraction blanks (2 per 96-well plate) were included to test for any possible contamination introduced during DNA extraction.

The DNA aliquots extracted from each of the 204 soil samples, their associated 6 extraction blanks, and 3 no-template controls were PCR amplified using a primer set that targets the hypervariable V4 region of the archaeal and bacterial 16S rRNA gene (515F [5′-GTGCCAGCMGCCGCGGTAA-3′] and 806-R [5′-GGACTACHVGGGTWTCTAAT-3′]) according to the methods described previously by Dragone et al. (29). These primers included the appropriate Illumina adapters and unique 12-bp barcode sequences to permit multiplexed sequencing (34). The amplified products of all samples, blanks, and no-template controls were cleaned and normalized to equimolar concentrations using SequalPrep normalization plates (Thermo Fisher Scientific, Carlsbad, CA, USA) and sequenced on an Illumina MiSeq run (Illumina, San Diego, CA, USA) using V2 2- by 150-bp paired-end Illumina sequencing kits.

The 16S rRNA gene sequences were processed using the DADA2 pipeline v.3.8 (35). Sequences were quality filtered and clustered into exact amplicon sequence variants (ASVs), with taxonomy determined using a naive Bayesian classifier method (36) trained against the SILVA reference database v.132 (37, 38). A minimum bootstrapping threshold required to return a taxonomic classification of 50% similarity was used for analysis. For the soil DNA extracts, ASVs associated with chloroplasts, mitochondria, and eukaryotes and those unassigned to the phylum level (717 ASVs) were removed prior to downstream analyses. We also excluded ASVs with fewer than 10 reads in total across the entire data set (1,567 ASVs). For our analysis, we used a cutoff of 1,000 reads per sample as a threshold for inclusion in our analysis. This left 167 samples that had a sufficient number of prokaryotic 16S rRNA gene reads for downstream analyses, with a mean number of reads per sample of 32,048 (range, 1,086 to 73,690) (Data Set S1B). We note that our blanks and negative controls did not show any evidence of contamination during the extraction or amplification steps (see reference 29 for more detail).

### Quantitative PCR.

To estimate how prokaryotic DNA concentrations vary across the sample set, we used quantitative PCR (qPCR) to measure bacterial 16S rRNA gene copy numbers using the same primers and soil DNA extracts used for sequencing. Reaction conditions and details were described previously (39). The 167 soil samples, corresponding extraction blanks, and 16 no-template controls were used for the 16S rRNA gene qPCR analyses. Standard curves were calculated using purified genomic DNA from Escherichia coli for 16S rRNA copy numbers. Based on the data from the negative controls, samples with a cycle threshold (*C_T_*) value of >31 were considered below detection limits. Calculated copy number measurements for each sample are reported as the number of E. coli genome equivalents per gram of soil.

### Microbial community analyses via marker gene sequencing.

Community analyses of the sequenced soil samples were performed in R v.4.0.5 (40). Richness was calculated from the filtered 16S rRNA gene ASV tables using specnumber (R package Vegan). Plots of relative abundance were created using the R package mctoolsr (https://github.com/leffj/mctoolsr/), as were the nonmetric multidimensional scaling (NMDS) plots. To measure differences between communities across the Shackleton Glacier region, we calculated pairwise Bray-Curtis dissimilarities from the ASV tables using the calc_dm function (R package mctoolsR). To identify the best model that explains the differences in overall prokaryotic community composition across the soil samples, we used BIOENV (41, 42) to identify the subset of biologically relevant environmental and geochemical variables that maximizes the correlation to Bray-Curtis dissimilarities (method, spearman). For these analyses, we included only samples where every variable was measured (108 soil samples). We then confirmed the correlation of each variable identified to the pairwise Bray-Curtis dissimilarities from the full set of 167 samples with Mantel tests. For all Mantel tests, distance matrices were calculated with the R function dist, and Mantel statistics are based on Pearson’s product-moment correlation method.

Phylogenetic tree construction was performed with the 100 most abundant bacterial ASVs. The phylogenetic relatedness of the 100 ASVs was determined via maximum likelihood with RaxML v.8.0.0 (raxmlHPC -f a -m GTRGAMMA -p 12345 -x 12345 -number 100 [43]), including Gemmata obscuriglobus as the outgroup. Sequences were aligned using MUSCLE (44), and the tree was visualized and annotated using iTOL v.6.3.2 (45).

### Network analysis and niche modeling of prokaryotic communities.

To identify modules of cooccurring prokaryotic ASVs across the 167 soil samples, we performed network analyses on the filtered ASV table generated from the culture-independent sequencing of DNA extracted from soil samples. We included all ASVs that, after the filtering steps described above, were found in at least 10 samples (3,710 ASVs). A correlation matrix was generated using the R function correlate (method, spearman). This matrix was filtered so that only positive correlations of >0.75 were kept. This left a final edge list of 4,274 correlations from 885 nodes. Network analyses were conducted and visualized using the R package igraph. Routes were generated from the node and edge lists with graph_from_data_frame, and community structures were found using cluster_louvain. From these network analyses, we found 88 modules of cooccurring prokaryotic ASVs, with each module containing between 2 and 154 ASVs (Data Set S1C).

We performed random forest analyses to determine which, if any, of the measured environmental and geochemical variables were the best predictors of where the 88 prokaryotic modules could be found across the Shackleton Glacier region. For the purpose of our modeling, a module was reported as being present in a sample if reads associated with any of the ASVs assigned to that module were present. For our random forest models, we used the R package rfPermute and performed a random forest analysis with 100 trees and three variables tested at each split to identify the most important predictors. Models were accepted if the percent variance explained was >10%, and 38 modules had predictive models that passed this threshold. For these models, the variable that most increased the mean standard error (MSE) was identified as the variable that was most predictive of where the taxa within each module were most likely to be found so long as that variable increased the MSE by at least 5% (*P < *0.05). Predictive soil and site variables were identified for 28 of the prokaryotic modules. To visualize these relationships, the average standardized relative abundance (Z score) of each of the 28 modules was plotted against the respective predictive variable. Z scores of each ASV were calculated from the filtered table of read counts using the R function zscore. The average standardized relative abundance of each module in each sample was calculated by averaging the Z scores of all ASVs assigned to that module.

### Metagenomic sequencing and annotation.

We chose 27 of the 167 samples for shotgun metagenomic sequencing (Table S2). This subset of samples was chosen to include at least two soil samples from each of 8 sampled features to span the range of edaphic properties found across our data set. We chose not to include samples from Schroeder Hill (SH) and Roberts Massif (RM) because the results of our amplicon sequencing effort suggested that we would not be able to extract enough DNA from these soils. To obtain sufficient DNA for metagenomic sequencing of the 27 samples, we reextracted DNA from these soil samples in triplicate using the Qiagen (Germantown, MD, USA) DNeasy PowerSoil kit. The manufacturer’s protocols were followed except that DNAs from all three replicates were combined on the same spin filter at the final step. This DNA was used to generate metagenomic libraries with the Nextera DNA Flex library preparation kit (Illumina, San Diego, CA, USA). The manufacturer’s protocol was followed except that the number of PCR cycles was increased for low-biomass samples as suggested previously by Bruinsma et al. (46) and by Illumina tech support. Libraries were sequenced on an Illumina NextSeq 500 run using a high-output 300-cycle kit with paired-end chemistry at the University of Colorado—Boulder’s Next-Generation Sequencing Facility.

10.1128/msystems.01330-21.9 TABLE S2 The 27 soil samples used for shotgun metagenomic sequencing. Download Table S2, JPG file, 1.0 MB. Copyright © 2022 Dragone et al. 2022 Dragone et al. https://creativecommons.org/licenses/by/4.0/ This content is distributed under the terms of the Creative Commons Attribution 4.0 International license.

Prior to downstream analyses, we removed adapter sequences from the raw sequence data using Cutadapt v.2.1 with the recommended options for paired-end Illumina reads (47) and filtered reads based on sequence quality using Sickle v.1.33 (-q 20 -I 50) (48). After this quality filtering, we obtained an average of 23.6 million quality-filtered reads per sample (range, 17.0 million to 28.4 million reads). The relative abundances and diversity of bacteria and archaea in the metagenomic samples were determined by extracting 16S rRNA gene reads from the metagenomic sequence data using phyloFlash v.3.0 (49). To verify that the 16S rRNA amplicon data were consistent with the taxonomic compositions of the bacterial communities as inferred from the metagenomic data, we tested the correlation between the Bray-Curtis dissimilarity matrices of the amplicon and metagenomic data sets using Mantel tests as described above (*r* = 0.80; *P* < 0.001).

Assembly-free analyses on the trimmed and quality-filtered data were performed using SqueezeMeta v.0.1.0 with the alternative analysis mode sqm_reads.pl script (50), which uses DIAMOND v2.0.11 (51) to annotate reads with the KEGG ontology (52–54). We obtained an average of 7.2 million annotated reads per sample across all 27 samples (range, 5.2 million to 8.7 million reads). To control for differences associated with variation in the number of annotated reads per sample, we rarified each sample to 5,203,694 annotated reads per sample using the R package vegan. This rarefied table was normalized using MUSiCC v.1.0.3 to obtain more robust measures of gene abundances normalized to the abundances of universal single-copy genes (55).

### Estimation of maximal microbial growth rates.

To estimate the maximal microbial growth rate, we used the tool gRodon, which estimates maximal microbial growth rates from codon usage biases in highly expressed genes, an indicator of selection for rapid growth (56, 57). Briefly, we assembled the sickle-filtered reads with MEGAHIT v.1.2.9 (preset, meta-large) (58) and mapped the filtered reads back onto the reference using Bowtie2 v.2.4.4 (default parameters) (59). We then used Metaprokka v.1.14.6 (https://github.com/telatin/metaprokka) to annotate the assembled reads. After annotation, we used the tool featureCounts (60) to calculate the number of filtered reads mapping to each gene and then converted these mapping counts to transcripts per million (TPM) (61, 62) to normalize for differential sequencing depths across samples and differences in gene length. We then ran gRodon (56) in metagenome mode to calculate codon usage biases between highly expressed ribosomal proteins and background codon usage. We also followed the authors’ recommendations for extremophiles and used the temperature setting to set a growth temperature of 0°C for all samples. We note that gRodon is not calibrated for the extremely low-temperature environments found in Antarctica, and maximal microbial growth rates should be considered estimates. For this reason, we focus on the relative comparison of estimated maximal growth rates between categories of samples instead of the specific values obtained.

### Analysis of metagenomic sequencing data.

Functional diversity was determined using the rarefied and MUSiCC-normalized KEGG table. To identify which annotated genes were more abundant at different elevations, we grouped the 27 soil samples into two different categories. “High-elevation” samples were those collected above 800 m (*n* = 15), while “low-elevation” samples were those collected below 800 m (*n* = 12). We chose 800 m as the separation between the two categories as no samples were collected from an elevation ±75 m of 800 m. This 150-m “gap” corresponds to the average elevation of this subset of 27 samples (853 m). Additionally, the group of 15 samples from above 800 m had significantly higher concentrations of nearly all measured geochemical variables than the group of 12 samples collected below 800 m (Fig. S7).

10.1128/msystems.01330-21.7 FIG S7 Comparison of the properties of the high-elevation and low-elevation samples selected for shotgun metagenomic analyses. High-elevation samples were collected from above 800 m (*n* = 15), while low-elevation samples were collected from below 800 m (*n* = 12). For all comparisons, the significance (*P*) (as determined by a Mann-Whitney U test) is inset in the plot. Download FIG S7, JPG file, 0.7 MB. Copyright © 2022 Dragone et al. 2022 Dragone et al. https://creativecommons.org/licenses/by/4.0/ This content is distributed under the terms of the Creative Commons Attribution 4.0 International license.

To identify differences in functional gene abundances between the high-elevation and low-elevation groups, we first compared KEGG richness (number of unique KEGGs in each sample) values using a Mann-Whitney nonparametric test using the R function wilcox.test. Next, we calculated the log_2_ fold change in average gene abundances across the two elevation categories for each KEGG according to methods described previously by Quackenbush (63). KEGGs were classified as being consistently more abundant at higher elevations if they were, on average, more than twice as abundant at higher elevations than at lower elevations (log_2_ fold change of less than −1). KEGGs that were identified as being more abundant at higher elevations were annotated based on the KEGG Orthology database gene catalogs (52–54).

To make predictions about potential functions that are more prevalent at higher-elevation sites, annotated genes were categorized into larger functional categories based on their locations in the KEGG pathway database and/or the KEGG BRITE database (52–54). We did not consider pathways that are associated exclusively with eukaryotic organisms (KEGG pathway categories “organismal systems,” “human diseases,” and “drug development” and BRITE categories “drugs,” “diseases,” and others associated with eukaryotic organisms). For the purposes of assigning a potential function, if a gene was associated with multiple pathways, it was included in both pathways. Pathways of interest were those identified as having at least 5 genes that were >2 times more abundant above 800 m (Fig. S5). We note that none of the pathways of interest were complete, with every gene being >2 times as abundant above 800 m.

### Targeted analysis of trace gas metabolism genes.

For more detailed, targeted analyses of genes related to trace gas metabolism, we followed an approach described previously (32). To summarize, the quality-filtered and trimmed paired-end reads (see above for more details) were searched for the presence of 10 metabolic marker genes related to trace gas metabolism using the blastx function of DIAMOND v.2.0.11 (51). These included CoxL, MmoX, PmoA, group 1c [NiFe]-Hyd, group 1d [NiFe]-Hyd, group 1f [NiFe]-Hyd, group 1h [NiFe]-Hyd, group 1l [NiFe]-Hyd, group 2a [NiFe]-Hyd, and group 3 [NiFe]-Hyd. More specifically, sequence reads were searched against protein sequences of these 10 genes downloaded from the Greening lab metabolic marker gene database v.1 (64) using a query coverage of 80%. According to the methods of Bay et al. (32), hits were kept if they had an identity threshold of 50% for the [NiFe]-Hyd genes or 60% for all others and a maximum E value threshold of 10^−10^. Reads per gene were divided by the total number of trimmed and quality-filtered reads and are reported in all downstream analyses as a proportion of the total reads. Differences between high-elevation soil samples and low-elevation soil samples were assessed using Mann-Whitney nonparametric tests as described above. Z scores were calculated using the R function zscore.

### Data availability.

The sequencing data generated from the soil samples can be accessed in the NCBI Sequence Read Archive under BioProject accession number PRJNA699250.
