# Supplementary figures and images for: Elevational Constraints on the Composition and Genomic Attributes of Microbial Communities in Antarctic Soils
Source: mSystems. 2022 Jan 18;7(1):e01330-21. doi: 10.1128/msystems.01330-21 (PMC8765064; doi:10.1128/msystems.01330-21)

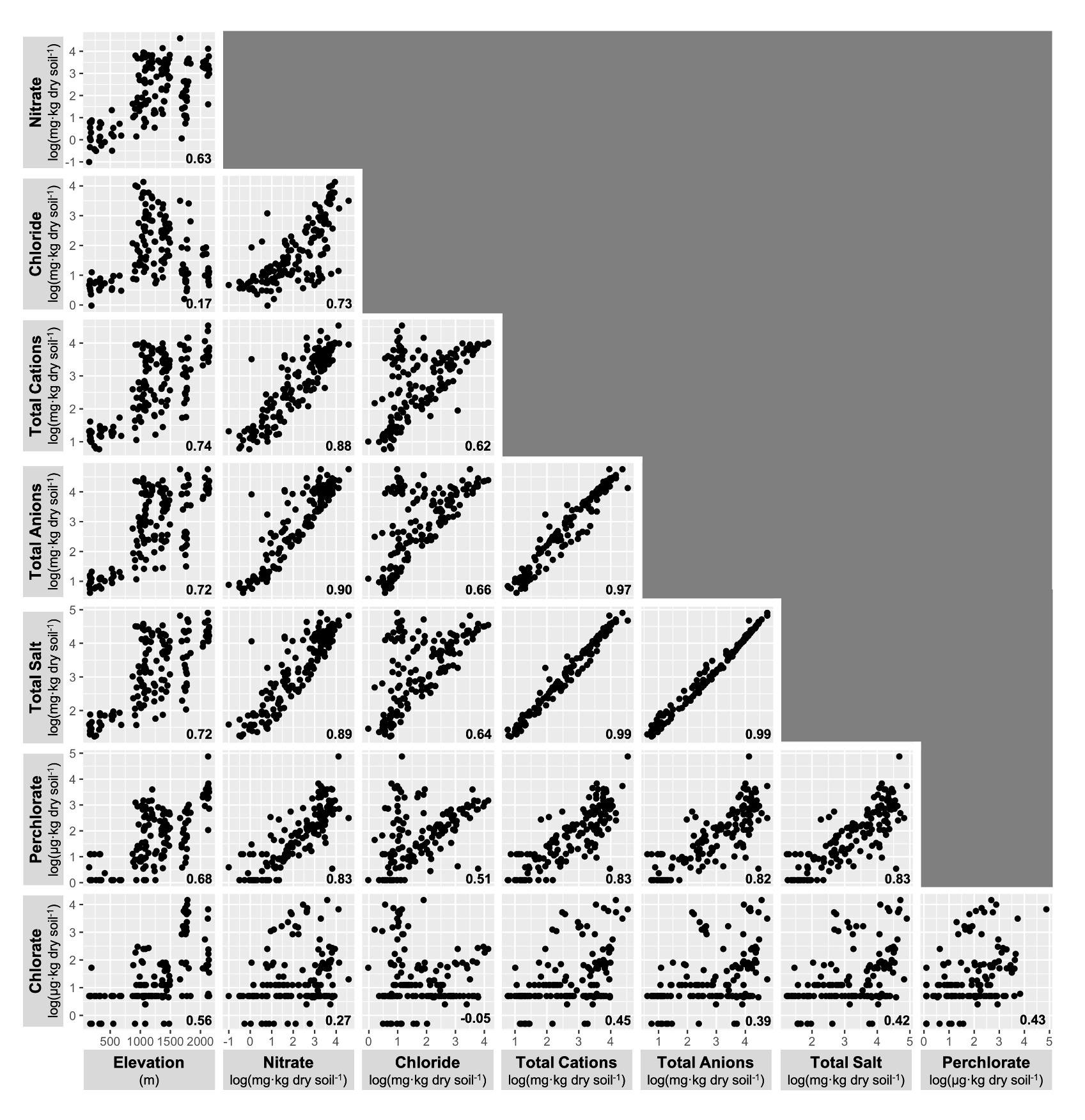

Supplement: FIG S1 [file msystems.01330-21-sf001.jpg]

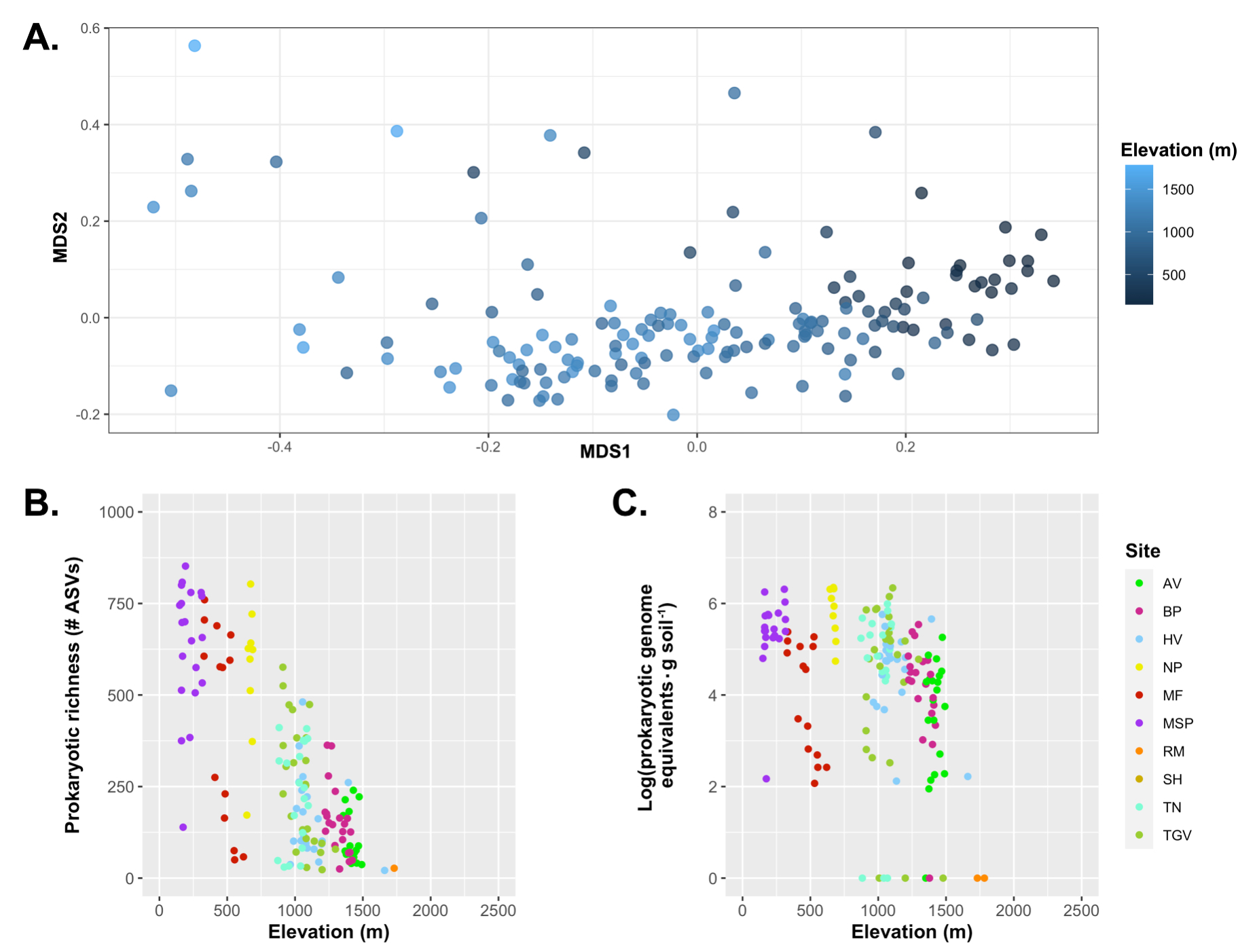

Supplement: FIG S2 [file msystems.01330-21-sf002.jpg]

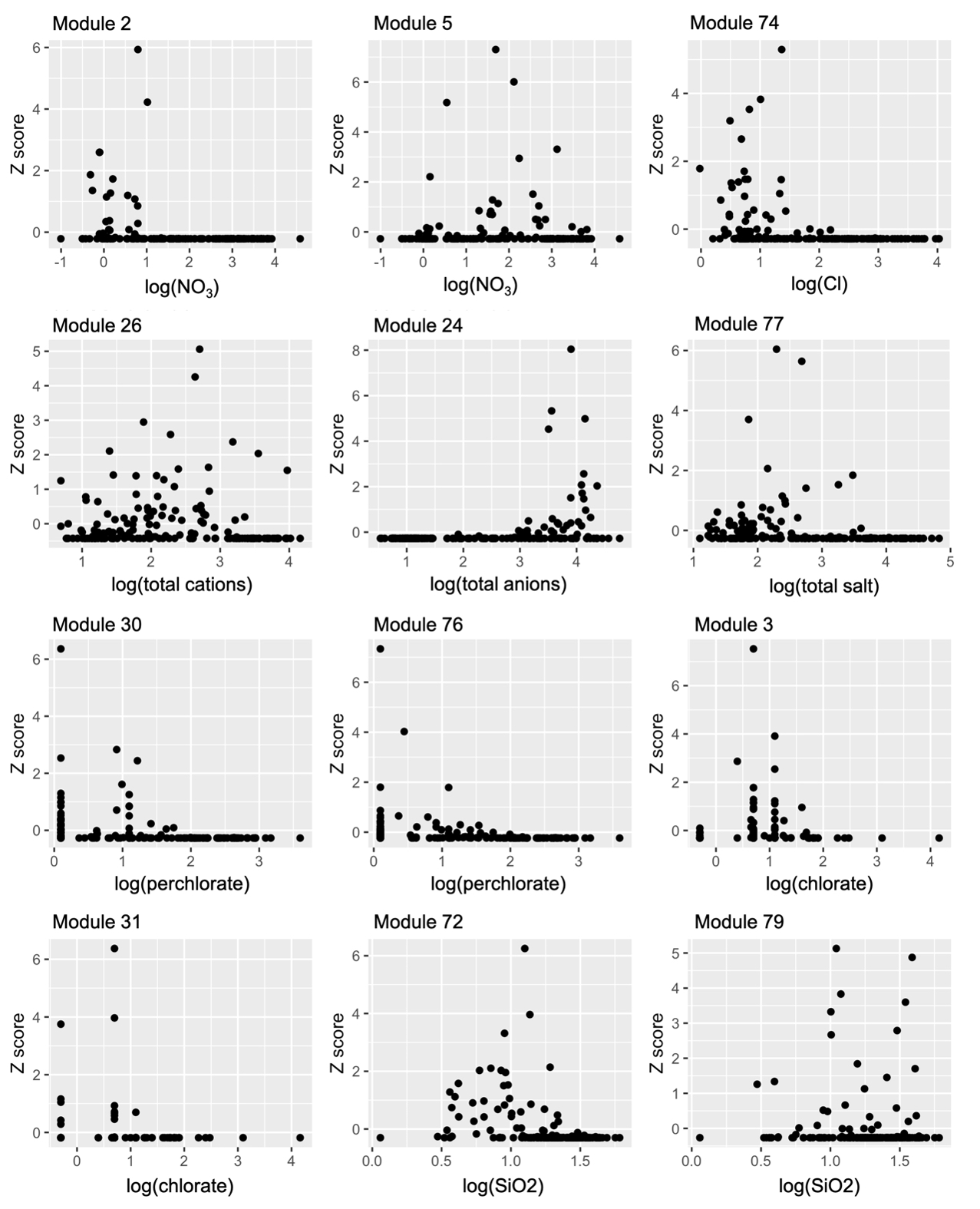

Supplement: FIG S3 [file msystems.01330-21-sf003.jpg]

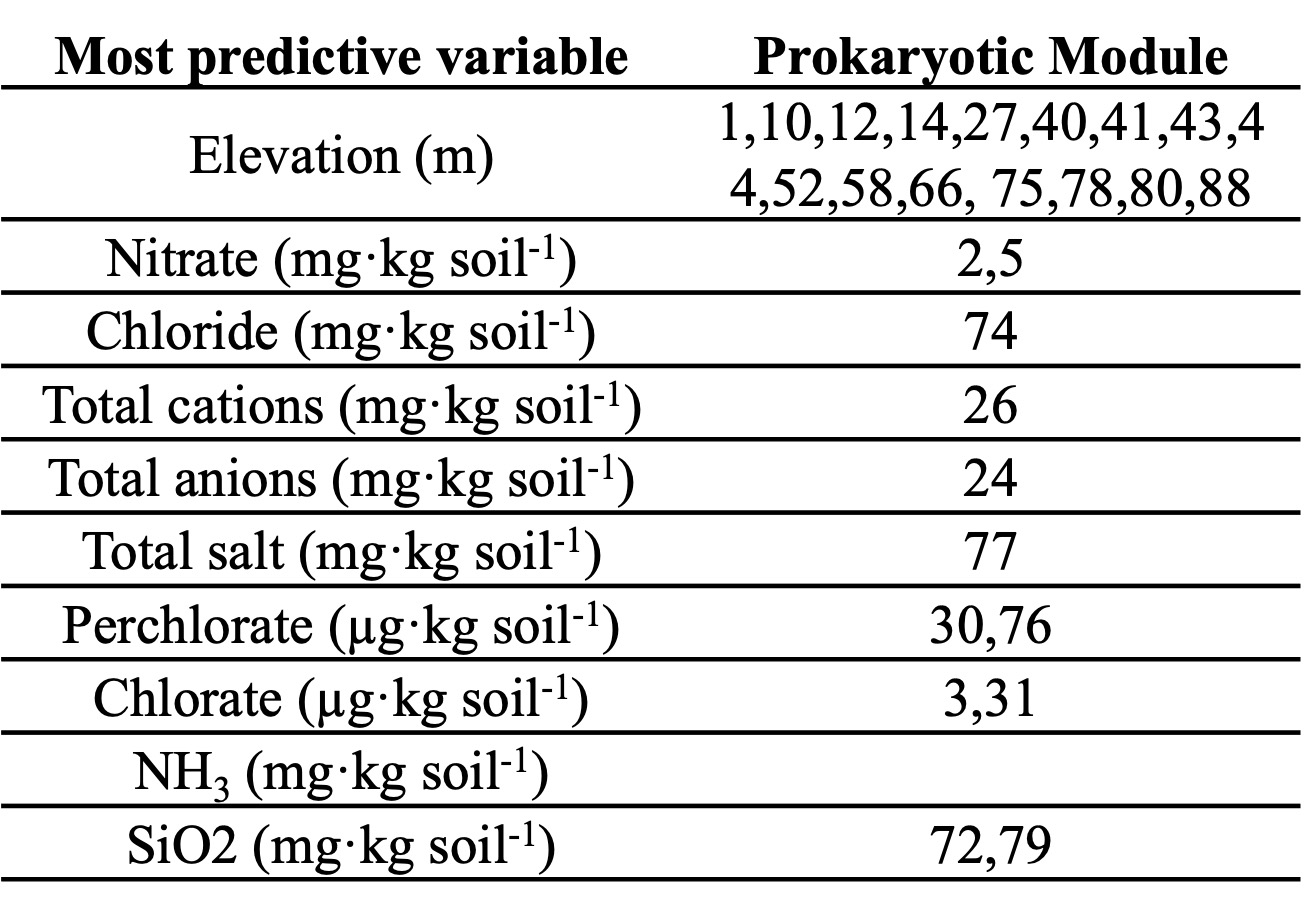

Supplement: TABLE S1 [file msystems.01330-21-st001.jpg]

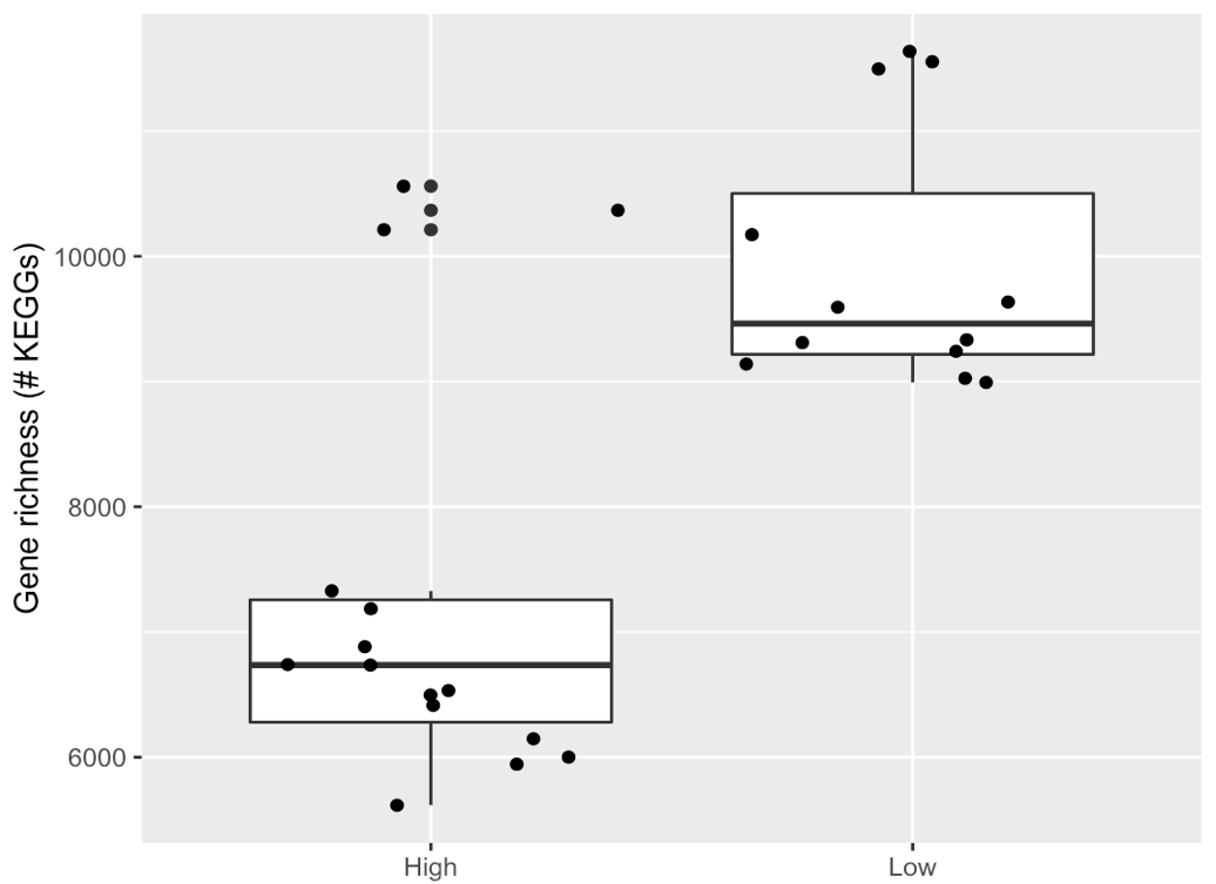

Supplement: FIG S4 [file msystems.01330-21-sf004.jpg]

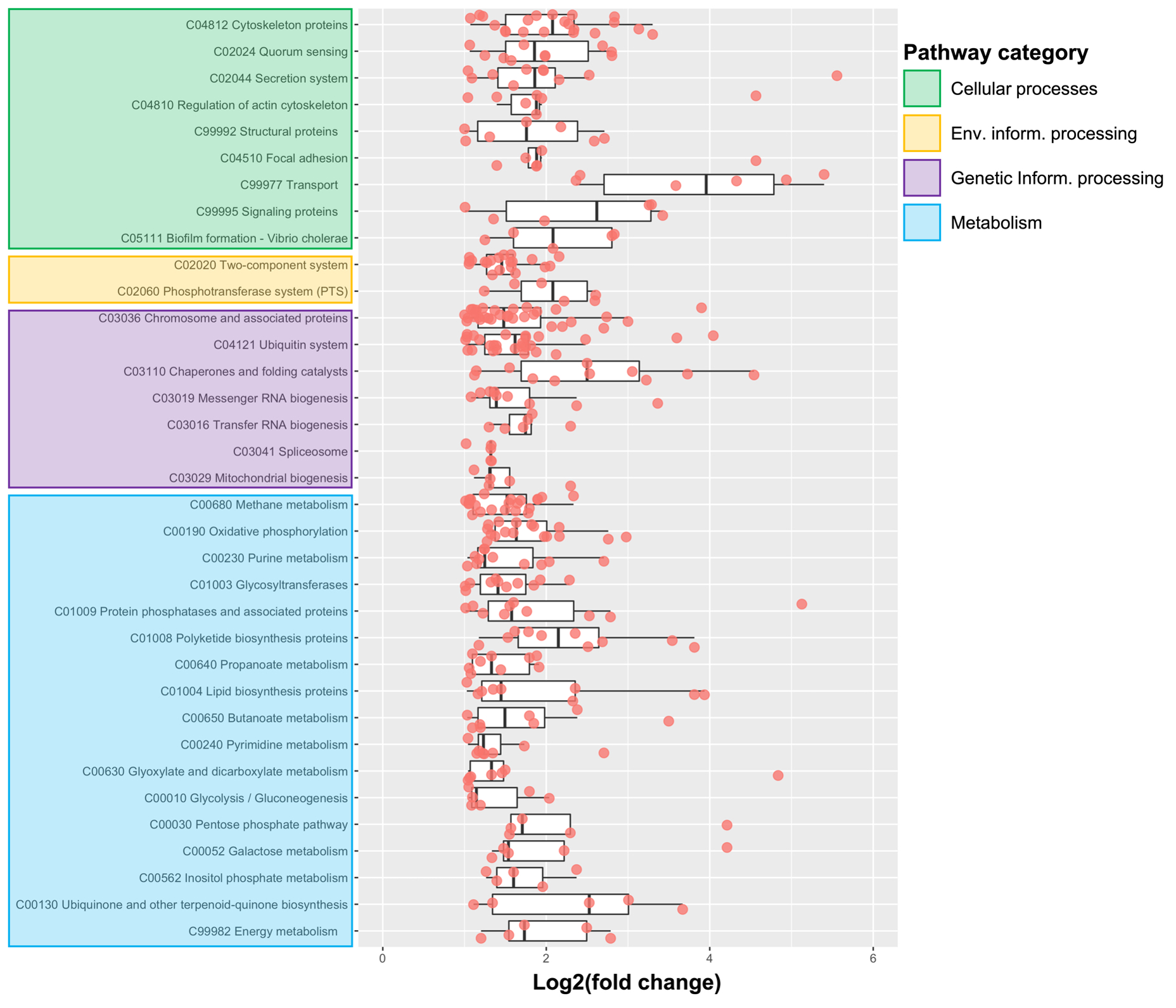

Supplement: FIG S5 [file msystems.01330-21-sf005.jpg]

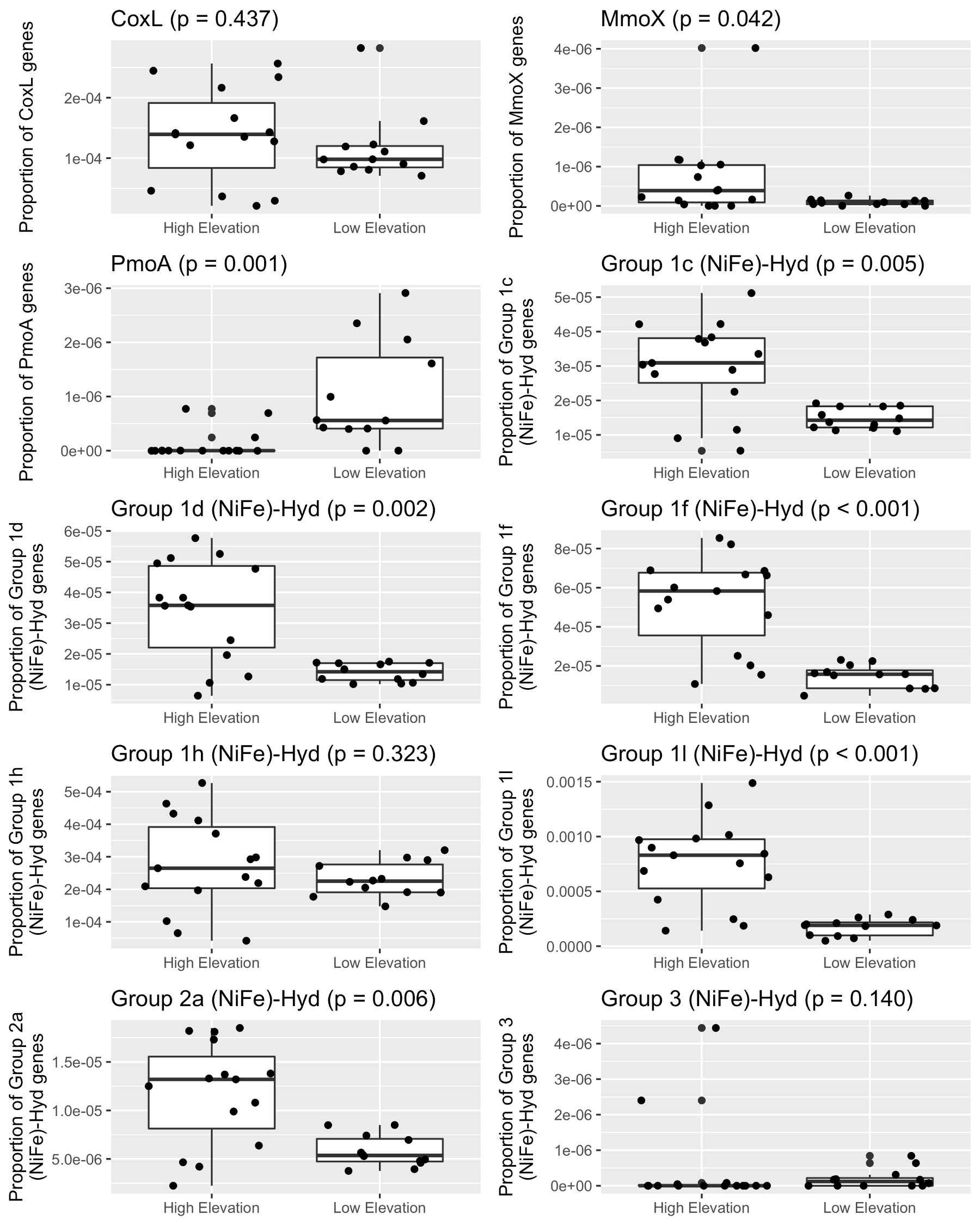

Supplement: FIG S6 [file msystems.01330-21-sf006.jpg]

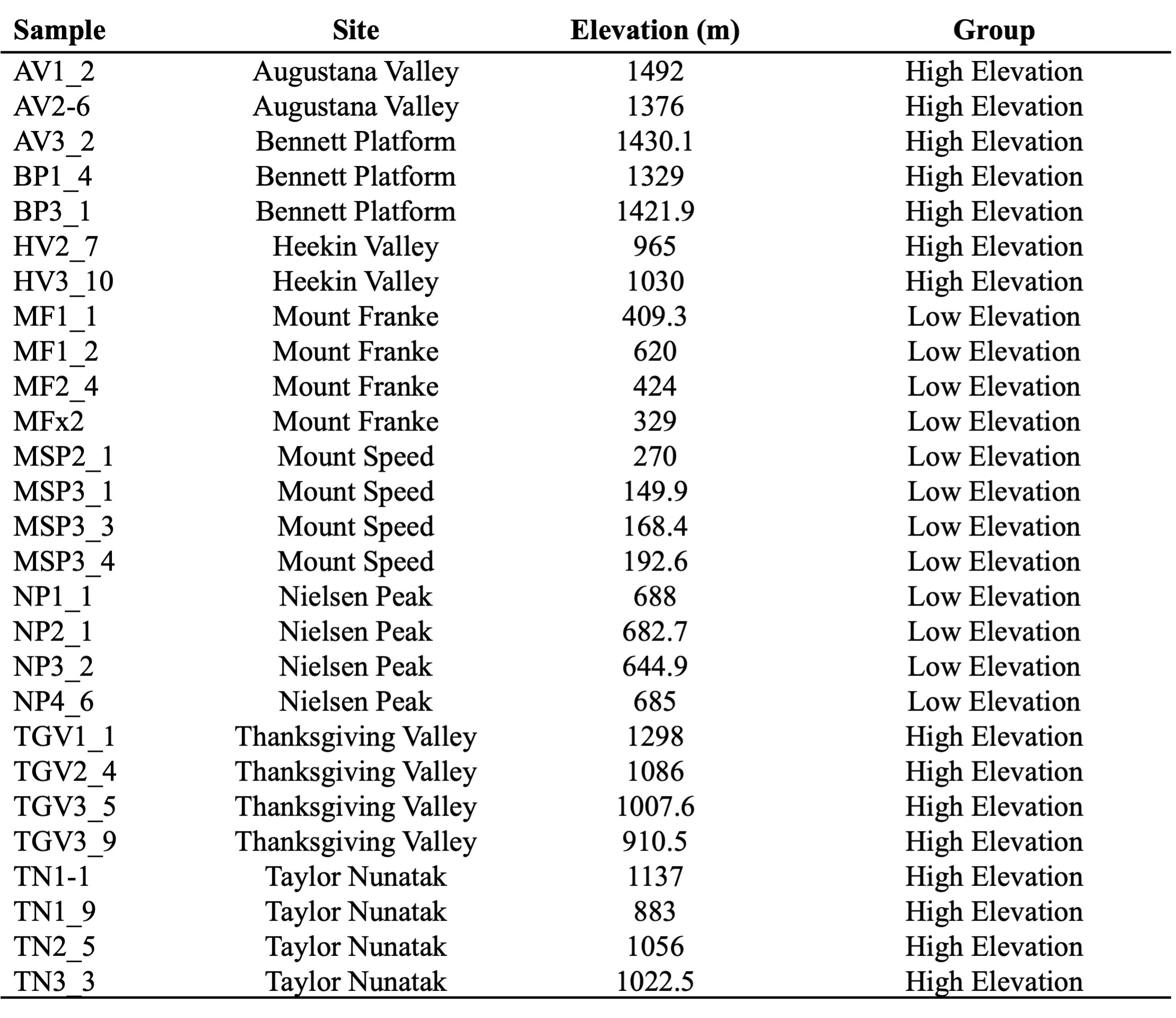

Supplement: TABLE S2 [file msystems.01330-21-st002.jpg]

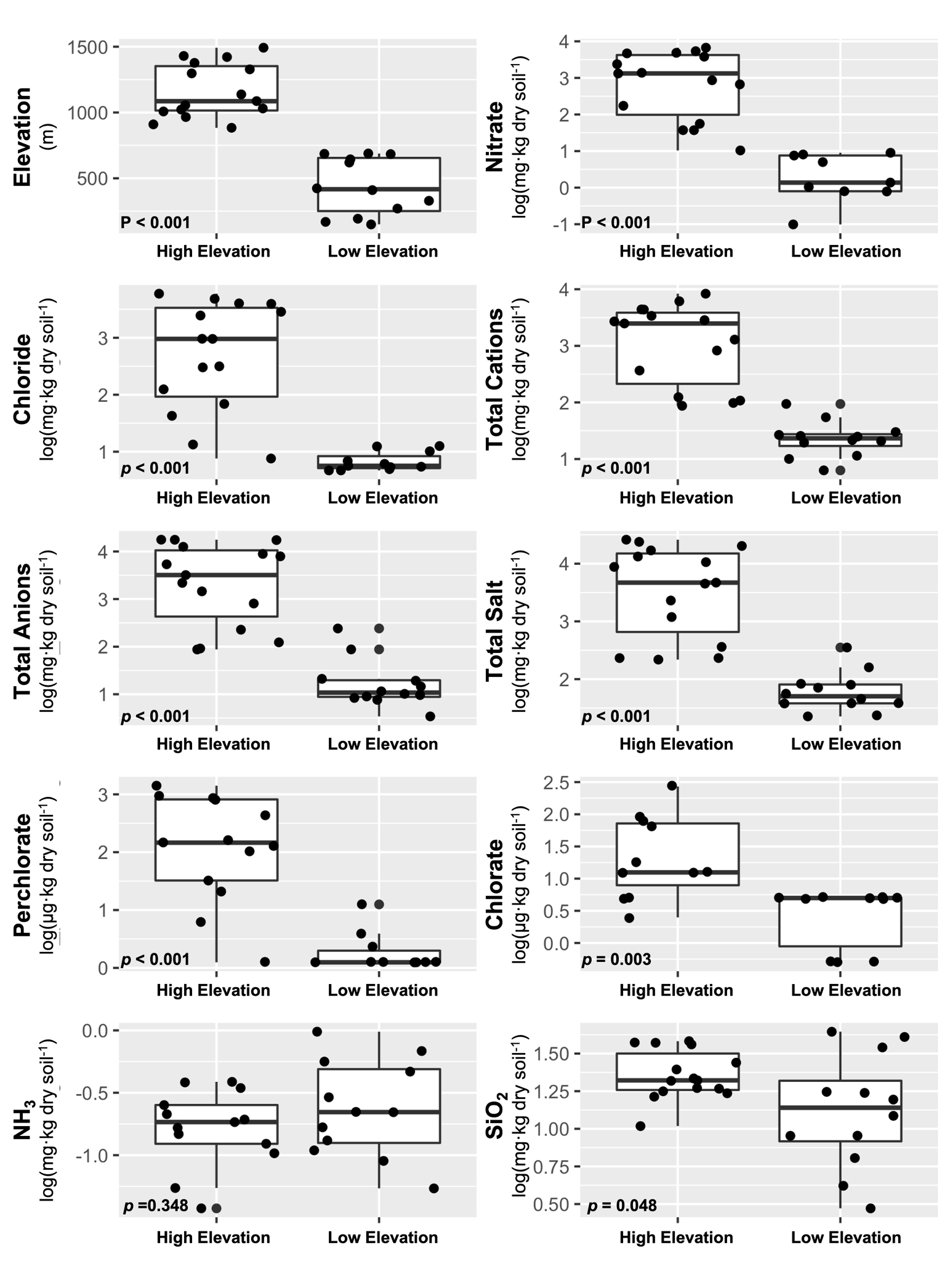

Supplement: FIG S7 [file msystems.01330-21-sf007.jpg]
